# Supplementary material for: Signatures of a time-reversal symmetric Weyl semimetal with only four Weyl points
Source: Nat Commun. 2017 Oct 16;8:942. doi: 10.1038/s41467-017-00938-1 (PMC5752680; doi:10.1038/s41467-017-00938-1)
Supplement: Supplementary file 1 — Supplementary Information [file 41467_2017_938_MOESM1_ESM.pdf]

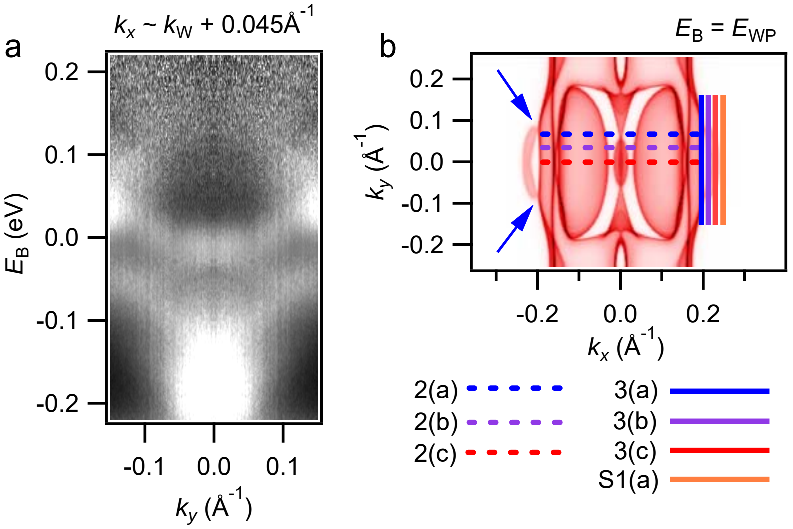

Supplementary Figure 1: **Systematics above E_F.** (a) Cut along k_y at $k_x \sim k_\mathrm{W} + 0.045\text{Å}^{-1}$, showing the Fermi arc receding into the upper Weyl cone, consistent with *ab initio* calculation and basic band theory, see also main text Fig. 3e. (b) Calculated Fermi surface above the Fermi level, at $E = E_\mathrm{W}$, with Weyl points marked by the arrows and the locations of the measured cuts marked by the solid and dashed lines.

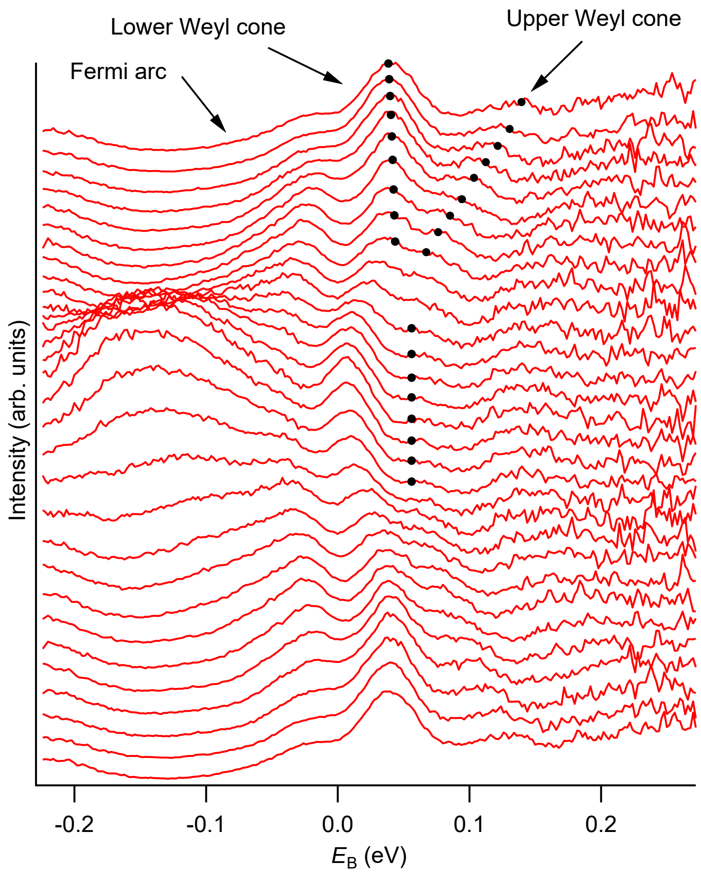

Supplementary Figure 2: **Systematic EDC stack at k_x approximately equal to k_W.** A systematic stack of energy distribution curves (EDCs) of the E_B-k_y cuts at $k_x \sim k_W$, Fig. 3a in the main text. The three trains of black dots indicate linear fits of three sets of peaks obtained by fitting EDCs and momentum distribution curves (MDCs). For the upper Weyl cone we plotted fits of the MDCs by Lorentzians, as illustrated in Fig. 3f of the main text. For the lower Weyl cone and the Fermi arc, we plotted fits of the EDCs by naive quadratics in the region of the peak. We can clearly follow the train of peaks. Near the band crossing it becomes difficult to perform the fit because there are several bands lying close together, so we omit this region from our fitting.

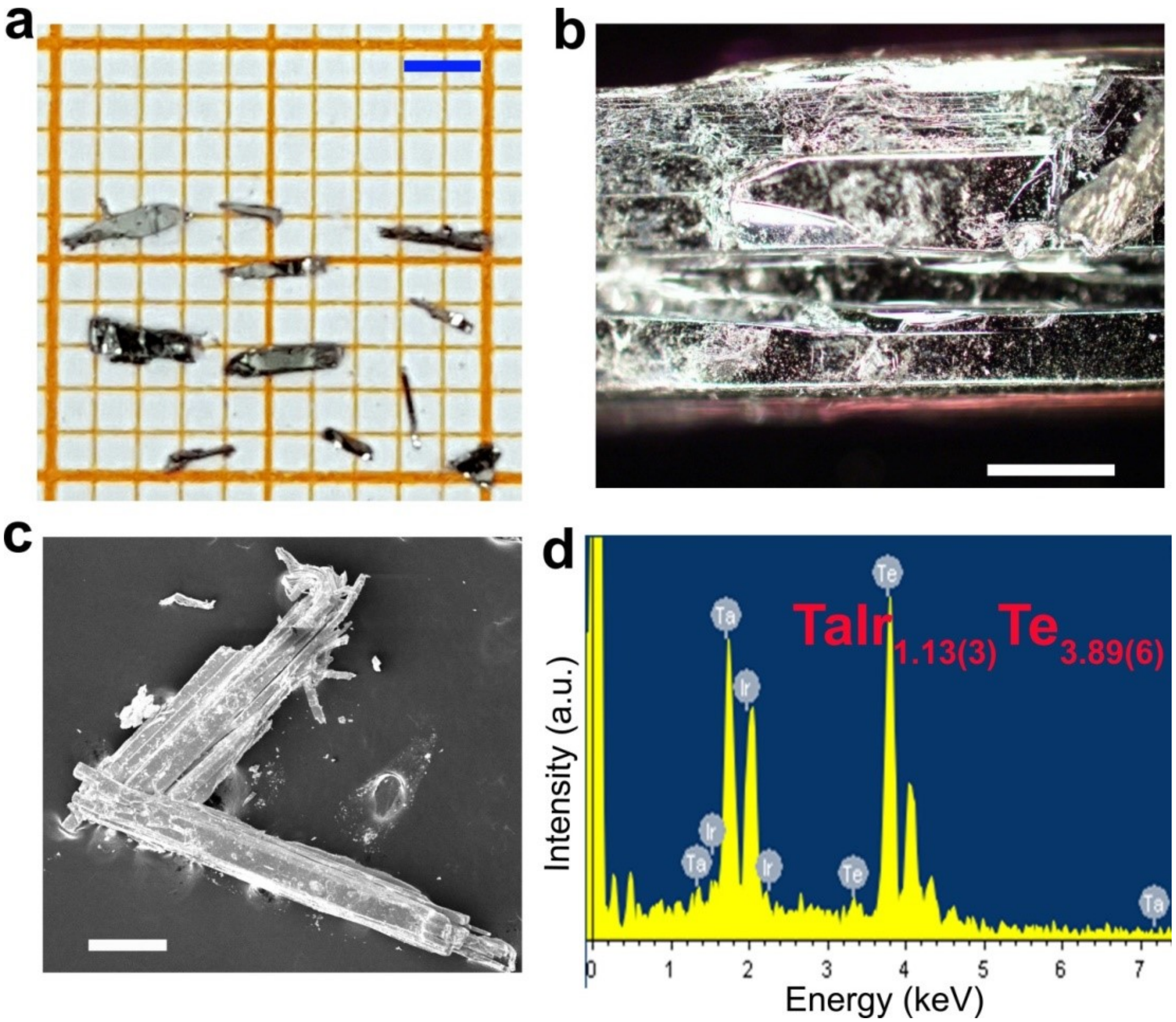

Supplementary Figure 3: **Characterization of $TaIrTe_4$ single crystals.** (a) Photo of as-grown $TaIrTe_4$ single crystals. (b) Optical micrograph of the surface of a $TaIrTe_4$ single crystal. (c) SEM image and (d) EDX spectrum of $TaIrTe_4$ single crystals, with the elemental ratio determined from the EDX spectrum. The scale bars are: (a) 2 mm, (b) 100 μm and (c) 200 μm.
